# Supplementary material for: COZOID: contact zone identifier for visual analysis of protein-protein interactions
Source: BMC Bioinformatics. 2018 Apr 6;19:125. doi: 10.1186/s12859-018-2113-6 (PMC5889581; doi:10.1186/s12859-018-2113-6)
Supplement: Supplementary file 2 — Software build. Executable binary file of the software tool. (ZIP 118,784 kb) [file 12859_2018_2113_MOESM2_ESM.zip › cozoid/platform/docs/testng-6.5.1-javadoc.zip!/org/testng/IInvokedMethod.html]

IInvokedMethod (TestNG)


---


|  |  |  |
| --- | --- | --- |
| **Overview** \| **Package** \| **Class** \| **Tree** \| **Deprecated** \| **Index** \| **Help** | |  |
| **PREV CLASS**   **NEXT CLASS** | **FRAMES**    **NO FRAMES**     **All Classes** |  |
| SUMMARY: NESTED \| FIELD \| CONSTR \| METHOD | DETAIL: FIELD \| CONSTR \| METHOD |  |


---


## org.testng Interface IInvokedMethod

---

``` public interface IInvokedMethod ```

An interface representing a method that has been invoked by TestNG.
This interface is internal.

---

| **Method Summary** | |
| --- | --- |
| `long` | `getDate()` |
| `ITestNGMethod` | `getTestMethod()` |
| `ITestResult` | `getTestResult()` |
| `boolean` | `isConfigurationMethod()` |
| `boolean` | `isTestMethod()` |

**Method Detail**

### isTestMethod

```
boolean isTestMethod()
```

:   **Returns:**: true if this method is a test method

---


### isConfigurationMethod

```
boolean isConfigurationMethod()
```

:   **Returns:**: true if this method is a configuration method (@BeforeXXX or @AfterXXX)

---


### getTestMethod

```
ITestNGMethod getTestMethod()
```

:   **Returns:**: the test method

---


### getTestResult

```
ITestResult getTestResult()
```

---


### getDate

```
long getDate()
```

:   **Returns:**: the date when this method was run


---


|  |  |  |
| --- | --- | --- |
| **Overview** \| **Package** \| **Class** \| **Tree** \| **Deprecated** \| **Index** \| **Help** | |  |
| **PREV CLASS**   **NEXT CLASS** | **FRAMES**    **NO FRAMES**     **All Classes** |  |
| SUMMARY: NESTED \| FIELD \| CONSTR \| METHOD | DETAIL: FIELD \| CONSTR \| METHOD |  |


---
